# Emergence of consistent intra-individual locomotor patterns during zebrafish development

**DOI:** 10.1038/s41598-019-49614-y

**Published:** 2019-09-20

**Authors:** Jennifer A. Fitzgerald, Krishna Tulasi Kirla, Carl P. Zinner, Colette M. vom Berg

**Affiliations:** 1 Eawag, Swiss Federal Institute of Aquatic Science and Technology, Department of Environmental Toxicology, Dübendorf, 8600 Switzerland; 2 Present Address: AstraZeneca, Patient Safety, Pepparedsleden 1, Mölndal, 43183 Sweden; 3 ETH Alumni Association, Rämistrasse 101, 8092 Zürich, Switzerland

**Keywords:** Animal behaviour, Neuroscience

## Abstract

The analysis of larval zebrafish locomotor behavior has emerged as a powerful indicator of perturbations in the nervous system and is used in many fields of research, including neuroscience, toxicology and drug discovery. The behavior of larval zebrafish however, is highly variable, resulting in the use of large numbers of animals and the inability to detect small effects. In this study, we analyzed whether individual locomotor behavior is stable over development and whether behavioral parameters correlate with physiological and morphological features, with the aim of better understanding the variability and predictability of larval locomotor behavior. Our results reveal that locomotor activity of an individual larva remains consistent throughout a given day and is predictable throughout larval development, especially during dark phases, under which larvae demonstrate light-searching behaviors and increased activity. The larvae’s response to startle-stimuli was found to be unpredictable, with no correlation found between response strength and locomotor activity. Furthermore, locomotor activity was not associated with physiological or morphological features of a larva (resting heart rate, body length, size of the swim bladder). Overall, our findings highlight the areas of intra-individual consistency, which could be used to improve the sensitivity of assays using zebrafish locomotor activity as an endpoint.

## Introduction

The ontogeny of zebrafish (*Danio rerio*) locomotor behavior and the underlying maturation of the locomotor network has been the subject of extensive studies, fueled by the vast variety of genetic, molecular, physiological and behavioral tools developed for this prominent vertebrate model organism. The first embryonic movements start around 17 hours post fertilization, but it is not until 2–3 days post fertilization (dpf) that larvae begin to swim spontaneously^1,2^. Initial movements are in short and infrequent bursts, eventually transitioning towards a beat-and-glide swimming mode after swim bladder inflation and before feeding at 5 dpf^3,4^. This sequence of events and the cellular mechanisms underlying this behavior are now well described in the literature^5–7^. As a result, the analysis of zebrafish locomotor activity has become a popular read-out to assess the impact of an external challenge to the nervous system for many fields of research. The amenability to high-throughput, non-invasive analyses which enables cost-, material- and time-efficient testing, compared to other vertebrate model organisms, has additionally contributed to the popularity of zebrafish behavioral assays. In addition, the availability of commercial plug-and-play systems (e.g. Noldus, ViewPoint or Loligo^®^ Systems) has simplified the acquisition and analysis of zebrafish locomotor data, making this an endpoint that can now be readily used. Despite such promising advances, zebrafish locomotor activity tests often suffer from high inter-individual variability. This ultimately impairs both the robustness and repeatability within which small, but potentially important, biological effects can be detected^8–14^.

Inter-individual variability in behaviors are widely observed in natural populations, including among humans^15,16^, birds^17,18^, fish^19^, and other species^20,21^. Such variability arises through genetic, developmental, pharmacological, environmental and social processes^22,23^ and play an essential role in defining the overall response and adaptation of a population to changes to its environment^24–26^. Such variability is nevertheless often overlooked when behavior is quantified as averages with associated dispersions, with individuals treated merely as replicates^27–30^. However, environmental change can affect the variation without changing the mean, with potential biological significance obscured when such variation is ignored. It is necessary, therefore, to address and understand differences between the behavior of individuals as it could facilitate the understanding of an individual’s response during environmental adaption^25,26^.

Inter-individual differences within a population are not the only source of variation within a behavioral experimental dataset. High variability within an individual’s own response can also contribute towards the variation in an experiment, with these intra-individual differences mainly attributed to ontogenetic and environmental effects^31–33^. While intra-individual consistency in behavior has been widely addressed in primates and rodents, aquatic models are less characterized in this regard, despite their increasing importance in behavioral trials^19,34,35^. The existence of intra-individual consistency in locomotor activity in early larval zebrafish stages would allow baseline measures of locomotor activities of all individuals prior to treatment to which effects can then be normalized. This would allow a better estimation of effects, especially if these are small, thereby increasing the sensitivity of such tests. In particular, the testing of acute effects on the nervous system, whether through exposure to toxicants, drugs, stressors or other perturbations would be improved.

Therefore, the aim of this study was to test whether consistent locomotor activity of an individual zebrafish emerges during larval development and, if so, under which conditions this occurs. Given that light conditions shape locomotor patterns differently^36–39^, we hypothesized that intra-individual consistency might vary under different light conditions. In addition, we tested whether consistency can be observed from stimulus-triggered activity responses and whether individual differences can be attributed to physiological or morphological features of the larvae.

## Results

### Locomotor behavior is most predictable in darkness

**Figure 1:**
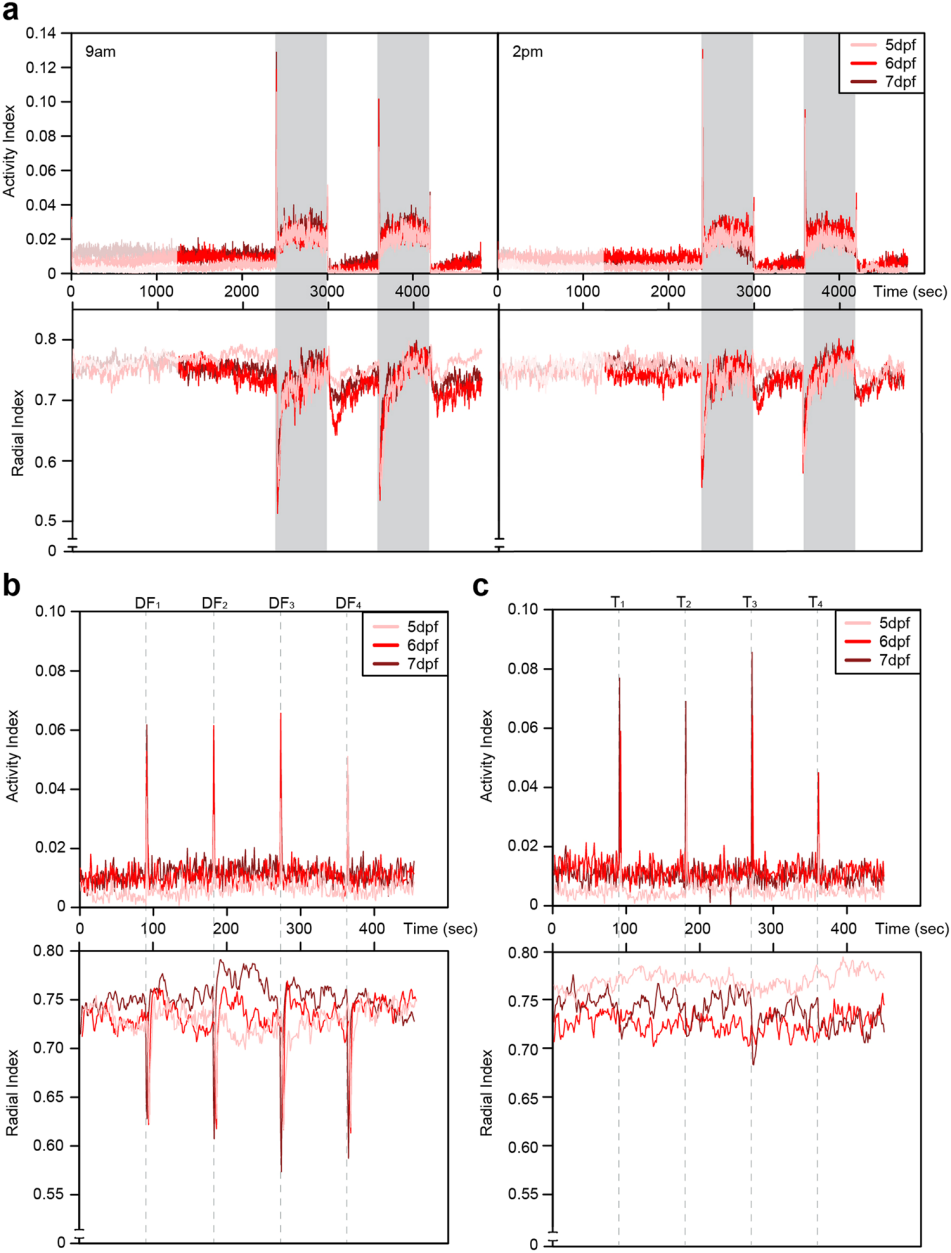
Time series plots from behavior experiments. (**a**) Average activity index (top) and radial index (bottom) per second of 132 zebrafish larvae over the study period under light or dark period at either 9 am (left) or 2 pm (right) for 5, 6 or 7 dpf. Initial 20 minutes acclimation period (faded period), followed by 20 minutes of “spontaneous” swimming, then swimming under darkness (2 × 10 min, referred to as “dark intervals”, shaded period) and swimming in light after periods of darkness (2 × 10 min, referred to as “light intervals”). For the startle triggers of (**b**) dark flashes (DF) and (**c**) tapping (T), average activity for 9 am measurements are shown. The dashed grey lines indicate occurrence of the stimuli.

To study the consistency of locomotor behavior of an individual larva over time, a total of 132 mixed wildtype (WM) larvae, derived from 3 replicates (Rep 1: n = 46, Rep 2: n = 41 and Rep 3: n = 45) were subjected to different behavior tests. Each test was performed at two time points (9 am and 2 pm) over three consecutive days (5, 6 and 7 days post fertilization, dpf; Supp. Fig. 1). The tests employed different lighting conditions, due to locomotor behavior of zebrafish larvae changing under different light conditions^36–39^. In each case, the following were analyzed: spontaneous swimming after a 20 min acclimatization period (referred to as “spontaneous”); swimming under darkness (2 × 10 min, referred to as “dark intervals”); and, swimming in light after periods of darkness (2 × 10 min, referred to as “light intervals”) (Fig. 1a). Figure 1a demonstrates the peaks of increased locomotor activity observed immediately after changes in lighting, as well as increased locomotor activity during dark intervals. In addition, we investigated within the same individual larvae whether their specific locomotor activity relates to their activity during startle responses, triggered firstly through four one-second dark flashes (Fig. 1b) and secondly, by using a tapping stimulus device integrated into the behavior system^40^ (Fig. 1c). We chose an inter-stimulus interval (ISI) of 90 seconds to measure the startle response of an individual repeatedly without inducing habituation^34^. Peaks of increased activity occurred immediately after application of each stimulus indicating that startle responses were triggered by these two protocols. To characterize swimming behavior during these tests, two metrics were calculated. Firstly the activity index, which is defined as the percentage of time an individual larva moves within one-second intervals. Secondly, the radial index, which indicates the location of a larva within the well and is calculated based on the distance of a larva from the center of the well, with large values representing proximity to the wall of the well and small values representing a more central location within the well. Although the activity and radial indices have been shown to be independent of one another^41^, our data indicates that the time at which an experiment is performed can influence this apparent independence (Supp. Table 1).

**Figure 2:**
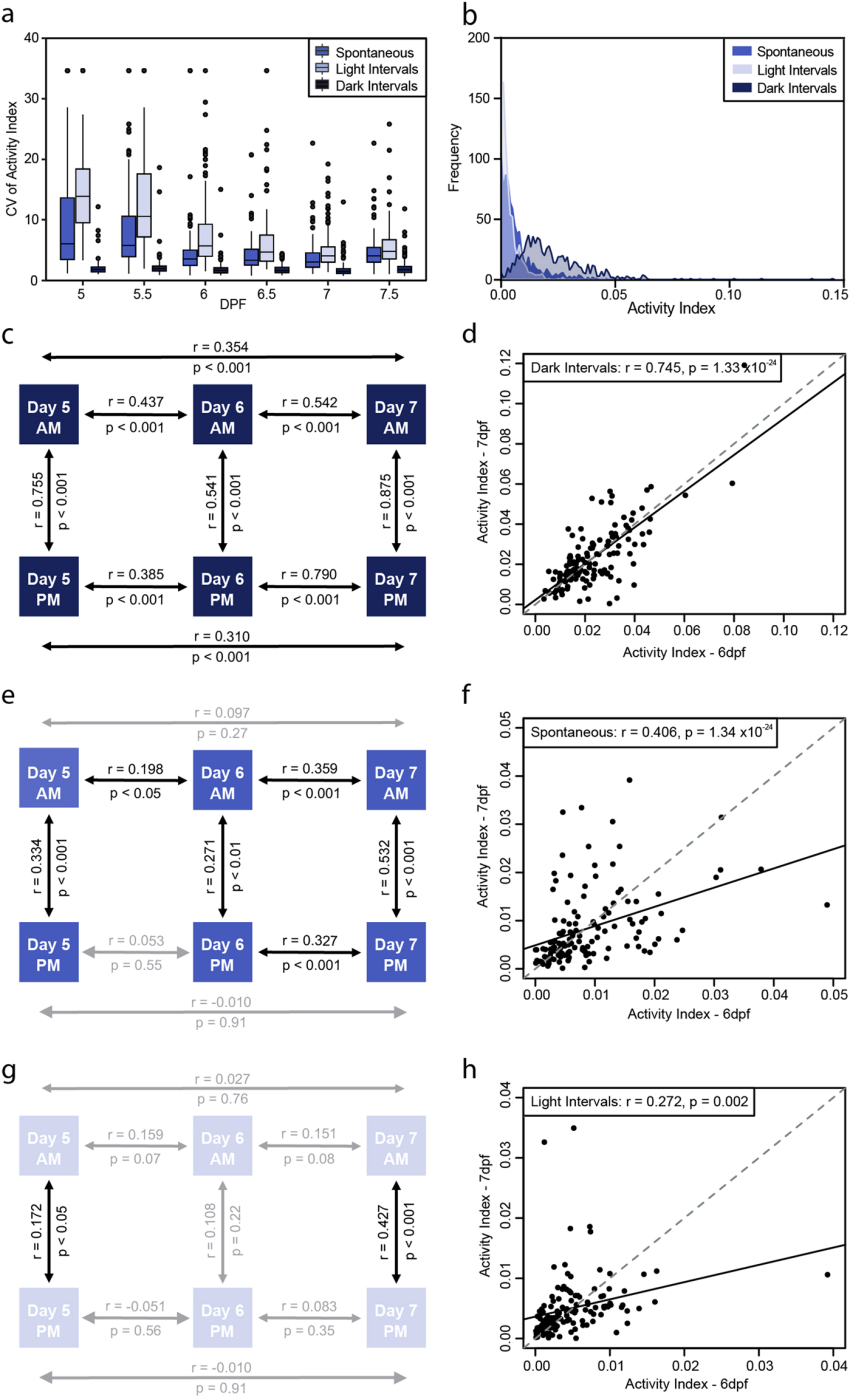
Behavioral intra-individual variability in a population of 132 larvae for the activity index. (**a**) Boxplot of the coefficient of variation of activity index for each individual larva (n = 132) over the different days (days post fertilization; dpf) under the different conditions studied. (**b**) Frequency distribution of the activity index in spontaneous, light and dark intervals depicting the differential activity profiles under these conditions. Schematics represent correlations of activity between different days and times of day for each of the conditions of study, (**c**) dark intervals, (**e**) spontaneous and (**g**) light intervals. Correlation plots between activity of larvae on day 6 vs 7 for (**d**) dark intervals, (**f**) spontaneous and (**h**) light intervals. Statistics on the plots represent the Pearson’s correlation coefficient and respective p value, with a linear regression line fitted for visual aid on the scatter plots (black solid line) and unity line (grey dashed line).

We found that intra-individual variability was consistently low during dark intervals, while during spontaneous swimming and light intervals it gradually decreased over development (Fig. 2a). Differences in activity distribution might explain these changes, as the activity of fish was lower during spontaneous and light intervals, versus dark intervals. Almost all larvae displayed either low or no activity during spontaneous and light intervals, while during dark intervals activity was comparatively increased (Fig. 2b). This is further supported by the fact that lower coefficient of variation (CV) values were generally associated with greater mean activity values, rather than a smaller standard deviation in dark intervals (Supp. Fig. 2a), indicating that the more that a larva moves, the less variable are its movements. By comparison, during the spontaneous swimming and light interval protocols, decreases in CV over time were found to correspond to higher mean activities with concurrently increased standard deviations (Supp. Fig. 2b,c).

Given that the intra-individual variability was found to be lowest during dark intervals, we analyzed whether an individual’s activity was consistently high or low under dark conditions during development by looking at the correlation of activity between the different days (5, 6, 7 dpf) and time of day (9 am and 2 pm). There was a strong correlation between measurements taken at 9 am compared to 2 pm for all days measured (5 dpf: r = 0.755, p < 0.001; 6 dpf: r = 0.541, p < 0.001; 7 dpf: r = 0.875, p < 0.001; Fig. 2c), indicating that there was no effect of time-of-day on a larva’s behavioral response to dark intervals. When looking at the different days, correlations were observed for all days (Fig. 2c) but the strongest correlations occurred between day 6 and 7 (r = 0.745, p < 0.001; Fig. 2d), suggesting that a larva-specific locomotor activity emerges at day 6 for each individual. Interestingly, activity during spontaneous swimming was less predictable (Fig. 2e), although a correlation remained during periods of spontaneous swimming, the magnitude of this correlation was weaker for days 6 and 7 (r = 0.406, p < 0.001; Fig. 2f) compared to the one observed in dark intervals (Fig. 2d). In addition, for light intervals, the interactions were even more unpredictable (Fig. 2g). Most comparisons resulted in no correlation and the correlation between day 6 and 7, although significant, was very weak (r = 0.272, p < 0.01; Fig. 2h), potentially due to the higher variation (Fig. 2a) and low activity the fish displayed (Fig. 2b).

To determine whether this increase in the strength of correlations over time is because of adaptation of the larvae to the experimental procedure or rather a developmental effect, we additionally tested whether the correlations would emerge earlier, i.e. between 5 dpf and 7 dpf, when starting the test at 4 dpf. No strong correlations could be determined when comparing 4 dpf to the other days, for any of the periods measured (Supp. Fig. 3). Additionally, no obvious improvement in the correlations between 5 dpf and 7 dpf were detected, suggesting that this lack of consistency in locomotor activity at earlier time points was due to developmental rather than technical/experimental effects.

**Figure 3:**
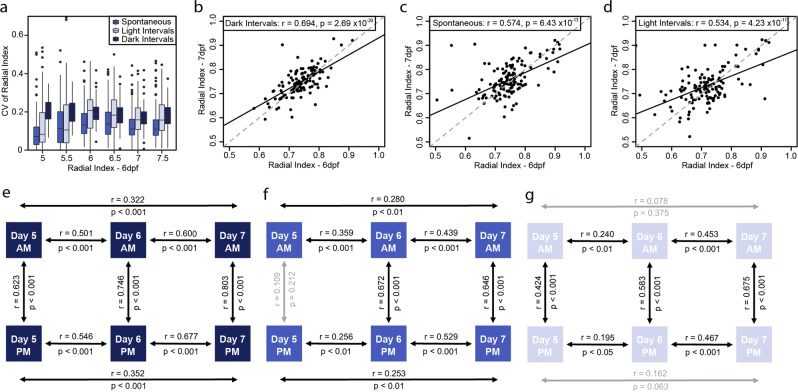
Behavioral intra-individual variability in a population of 132 larvae for the radial index. (**a**) Boxplot of the coefficient of variation of radial index for each individual larva (n = 132) over the different days (days post fertilization; dpf) under the different conditions studied. Correlation plots between the radial index of larvae on day 6 vs 7 for (**b**) dark intervals, (**c**) spontaneous and (**d**) light intervals. Schematics represent correlations of the radial index between different days and times of day for each of the conditions of study, (**e**) dark intervals, (**f**) spontaneous and (**g**) light intervals. Statistics on the plots represent the Pearson’s correlation coefficient and respective p value, with a linear regression line fitted for visual aid on the scatter plots (black solid line) and unity line (grey dashed line).

When considering the radial index, i.e. the location of larvae in the well, less intra-individual variability occurs between the different experimental conditions tested, compared to the activity index (Figs 2a and 3a). This indicates that larvae move throughout the entire well area, with some individuals demonstrating a higher tendency to swim closer to the wall (large radial index) than others (small radial index), independent of the applied lighting conditions (Fig. 3b–d). Interestingly, between the different days and time points there is a larger proportion of significant interactions for the radial index compared to the activity index (Fig. 3e–g), albeit the actual strength of the correlation tends to be weaker for the radial index (e.g. for dark intervals- radial index: r = 0.694, p < 0.001; Fig. 3b, activity index: r = 0.745, p < 0.001; Fig. 2d). This demonstrates that the radial index was less able to predict the movement between the two days compared to the activity index.

In summary, we show that although larval activity levels differed slightly between morning and afternoon, individual larvae move consistently throughout development, i.e. individuals moving more in the morning were also found to move more during the afternoon. Furthermore, under darkness, where zebrafish larvae are known to demonstrate elevated activity (Fig. 1a), intra-individual variability in both the activity and radial indices was reduced, becoming consistent by day 6.

### Startle responses are not consistent for an individual

**Figure 4:**
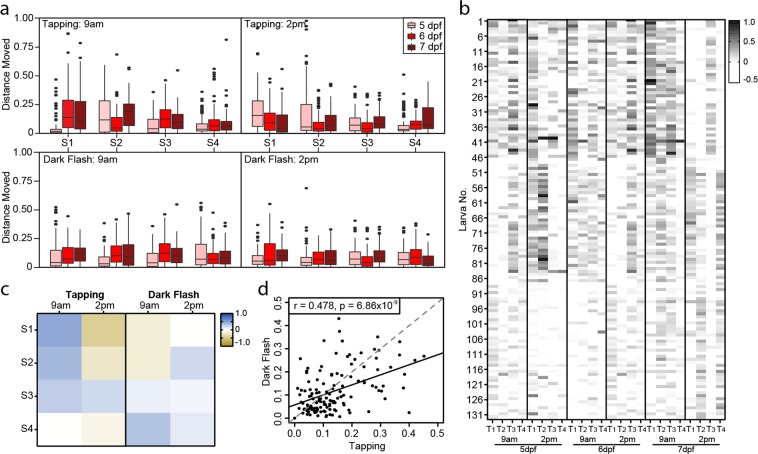
Individual responses to startle stimulus. (**a**) Boxplots of the average distance moved of the 132 larvae at each of the four stimuli (S1–S4) (tap or dark flash) at either 9 am or 2 pm. The different color boxes represent the three different days measured on 5, 6 and 7 days post fertilization (dpf). (**b**) Heat map representing the change in distance moved with respect to the baseline of each individual larva for all time points and days measured in response to each of the tapping stimulus (T1–T4). White represents no response to the stimulus, with the grey scale darkening in a linear scale depending on the strength of the response. (**c**) Heat map of the r values from the correlations of distance moved of individual larvae between 6 and 7 dpf, for each of the four startle stimuli at either 9 am or 2 pm for tapping and dark flashes. Blue represents a positive correlation, with yellow representing a negative correlation. (**d**) Correlation plot between the individual fish response to the first dark flash and the first tap at 6 dpf. Each point on the graph represents an individual larva (n = 132) and the correlation coefficient was calculated using Pearson’s correlation, with a linear regression line fitted for visual aid (black solid line) and unity line (grey dashed line).

We tested whether the locomotor activity of an individual zebrafish larva, exposed to different lighting conditions related to its activity during a startle response by measuring both behaviors within the same individuals (Supp. Fig. 1). Startle responses were triggered from two different protocols (“dark flash” and “tapping”) (Fig. 1b,c), as well as induced when switching the lights on (“onset”) and off (“offset”) (Fig. 1a), while locomotor activity was measured during spontaneous swimming and in light and dark intervals, as described above, using the activity index metric. The strength of startle responses was measured by calculating the distance moved during the one second following application of the stimulus. This was used instead of the activity index to better represent the full spread of the data, which was masked due to the small periods where data was collected. Similar outcomes were found for both measures (Supp. Table 2). The average responses of the 132 larvae to each of the four stimuli showed some differences, however, no clear trend was discernible (Fig. 4a). In addition, the individual responses showed neither a consistency nor a uniform decrease in strength across the different stimuli for both tapping (Fig. 4b) and dark flashes (Supp. Fig. 4), suggesting that fish are not consistently habituating to the stimuli. Indeed, when calculating a habituation index (HI) over the four stimuli, habituation occasionally occurs, but is not consistent for an individual over development (Supp. Fig. 5).

Looking closer at individual variability, we found a significant, albeit only moderate correlation when comparing the response from the first tapping stimulus between 6 and 7 dpf at 9 am (r = 0.423, p < 0.001; Fig. 4c). However, for responses in the afternoon this correlation becomes negative (r = −0.404, p < 0.001; Fig. 4c), potentially due to the larvae reducing their activity at 2 pm compared to 9 am, which is supported by the comparison of the average distance moved between those periods (7 dpf 9 am average distance moved: 0.138 ± 0.20; 7 dpf 2 pm average distance moved: 0.041 ± 0.11; p < 0.001; Fig. 4a). The significance of this correlation weakens with each tapping stimulus and is ultimately lost (Fig. 4c). Interestingly, this trend was not observed for the dark flash stimulus, where no significant correlations between the response of the fish on day 6 to 7 for either 9 am or 2 pm, irrespective of the stimulus number, were detected (Fig. 4c). The same inconsistency was found for responses triggered at onset and offset of light switches (Supp. Table 3).

When cross-comparing responses to tapping stimuli and dark flashes, we found a weak correlation for the first stimulus at day 6 (r = 0.478, p < 0.001; Fig. 4d). However, this was not consistently observed for all days or time points studied (Supp. Table 3), supporting further that the larvae’s response to a startle stimulus is not predictable or consistent at the individual or population level. Furthermore, when comparing an individual’s locomotor activity with startle response strength, no strong correlations were found for the different protocols used under all light conditions (Supp. Table 4), suggesting that the beat-and-glide swim mode does not relate to an individual’s startle response capabilities, irrespective of the stimulus modality.

### Locomotor activity is not associated with physiology and morphology

**Figure 5:**
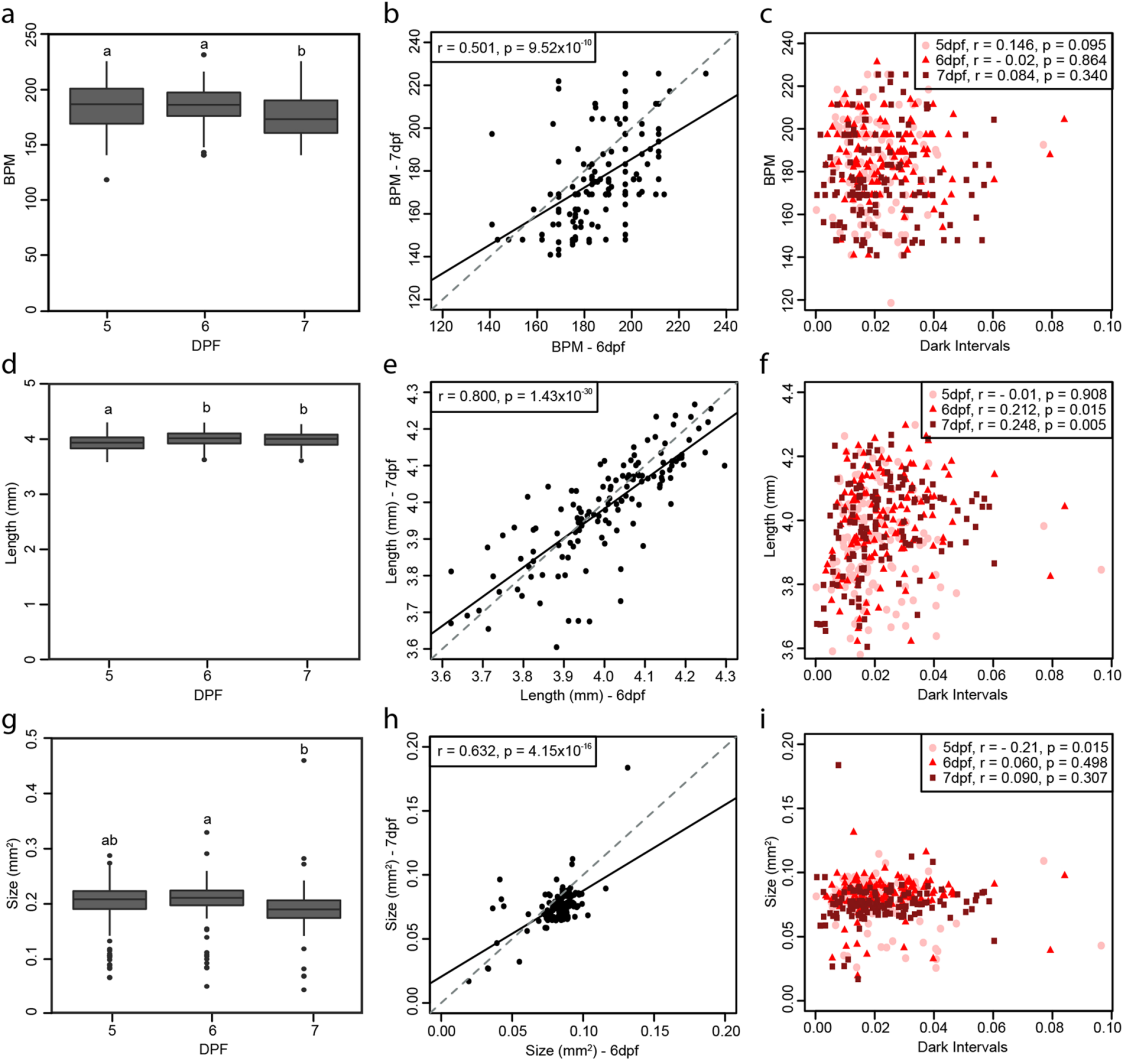
Physiology and morphometric parameter comparisons. Boxplots representing the average measure of all 132 larvae of (**a**) heart rate (beats per minute; BPM) (one-way ANOVA: F_(2, 393)_ = 9.73, p < 0.001 followed by Tukey HSD multiple comparison: p _(5dpf-6dpf)_ = 0.92, p _(5dpf-7dpf)_ < 0.01, p _(6dpf-7dpf)_ < 0.001), (**d**) body length (mm) (one-way ANOVA: F_(2, 393)_ = 54.328, p < 0.001 followed by Tukey HSD multiple comparison: p _(5dpf-6dpf)_ < 0.001, p _(5dpf-7dpf)_ < 0.001, p _(6dpf-7dpf)_ = 0.99) and (**g**) size of swim bladder (mm^2^) (one-way ANOVA: F_(2, 393)_ = 5.31, p < 0.01 followed by Tukey HSD multiple comparison: p _(5dpf-6dpf)_ = 0.255, p _(5dpf-7dpf)_ = 0.215, p _(6dpf-7dpf)_ < 0.01), over the three days of experiments (5, 6 and 7 days post fertilization, dpf) with significant difference represented by different letters on each graph. Correlation plots between 6 and 7 dpf for (**b**) heart rate, (**e**) body length and (**h**) size of swim bladder, with each point representing a single larva. Comparison of the individuals’ (**c**) heart rate, (**f**) body length and (**i**) size of swim bladder to their respective average activity during dark intervals, with each day plotted on each plot (5 dpf: circle, 6 dpf; triangle and 7 dpf; square). Statistics on the plots represent the Pearson’s correlation coefficient and respective p value, with a linear regression line fitted for visual aid on the scatter plots (black solid line) and unity line (grey dashed line).

Resting heart rates in individual larvae can vary. We tested if this property links to their inherent locomotor activity by measuring the heart rate of each individual immediately following the afternoon behavior experiments (Supp. Fig. 1). The resting heart rates of the WM larvae used in the behavioral experiments lay within a broad range of 118.60–225.46 beats per minute at 5 dpf with a mean of 185.55 BPM (±21.94). The mean did not significantly change at 6 dpf (mean = 186.51 ± 16.17), but at 7 dpf there was a significant decrease compared to day 5 and 6 (mean = 176.63 ± 21.58, p < 0.001; Fig. 5a). Despite this, the resting heart rate of an individual was significantly consistent over the three days measured (Fig. 5b, Supp. Table 5). However, although the resting heart rate and locomotor activity for an individual are consistent during development, they did not correlate during dark intervals (5 dpf: r = 0.146, p = 0.10; 6 dpf: r = −0.015, p = 0.86; 7 dpf: r = 0.084, p = 0.34; Fig. 5c) or for the other light conditions tested (Supp. Fig. 6a,d).

We further assessed morphological features of the larvae and tested whether they are consistent over time for each individual and whether they would underlie the differences in locomotor activity among the individuals. The mean body length of all 132 larvae was significantly smaller at day 5 compared to the other days, but there was no significant increase from day 6 to 7 (Fig. 5d). The body lengths strongly correlated between the days tested, suggesting that the individuals grow at a constant pace (Fig. 5e, Supp. Table 5). Again, during dark intervals, there was no strong correlation between activity of the larvae and their body length (Fig. 5f), with similar patterns observed for spontaneous movement and light intervals (Supp. Fig. 6b,e). Similarly, the size of the swim bladder was consistent for an individual over the 3 day experiment (Fig. 5h, Supp. Table 5). However, on average, swim bladders appeared significantly smaller on day 7 compared to day 6 (Fig. 5g). There was no significant correlation between the activity of larvae under dark intervals and the size of the swim bladder, although for day 5 there was a slight trend towards fish with smaller swim bladders moving more (Fig. 5i), with a similar trend also seen at 6 dpf for spontaneous movement and light intervals (Supp. Fig. 6c,f).

## Discussion

Behavioral diversity among a population can be observed throughout the animal kingdom, both in genetically diverse and in isogenic populations^42,43^. Behavioral inter-individual variability, as well as inter-strain variability, are increasingly reported for laboratory strains of zebrafish kept for long periods of time^44–49^. Besides genetic diversity, developmental^50,51^, maternal^52,53^ and epigenetic effects^41,54,55^, as well as social interactions^56^, are all thought to contribute to such variation. Recently, microRNAs (miRNAs) have been shown to play a role in regulating some behaviors in *Drosophila*^57–59^ and zebrafish^60^, potentially providing another source of behavioral variation. Despite this, variation among individuals is often overlooked when behavior is quantified as averages with associated distributions^24,27–30^. Therefore, in this study, we aimed to address the hypothesis that despite the high inter-individual variability in zebrafish locomotor activity, intra-individual consistency might emerge during larval development, possibly shaped by different light conditions and physical properties of the larvae.

Our data shows that locomotor activity begins to become predictable for an individual during development around 6 dpf, especially during darkness-induced explorative behavior. We also demonstrate that locomotor activity does not correlate with physiological and morphological features in larval stages, although these features are consistent within an individual. Our data additionally suggests that the consistency of an individual’s locomotor behavior is not linked to a specific genetic background of an inbred wildtype strain but can be observed in mixed wildtype larvae with genetically more diverse backgrounds. These conclusions are supported by previous research showing that swimming behavior is predictable between individuals when swimming freely in identical wells^41^. Roman *et al*. identified a histone H4 acetylation pathway that modulates individual behavior in a genetically-independent manner, without affecting the global average behavior of the population. Therefore, while average behavior might mostly depend on genetic background or changes in environment, inter-individual behavioral variability could result from differences in histone H4 acetylation.

The most consistent period for all behavior parameters measured was between 6 and 7 dpf, with the most predictable activity under dark intervals, as compared to spontaneous and light intervals. Spontaneous locomotion in zebrafish larvae is shown to follow a non-random pattern even in the absence of sensory cues, facilitating the detection of resources or shelter^61^. Driven by the dwindling nutritional stock supplied from the yolk, developing larvae start actively hunting for food from around 4 days post fertilization^62^. This predation is strongly dependent on a functional visual system, as larvae in darkness or with impaired vision are unable to locate prey^63,64^. Accordingly, upon change of light conditions, larvae engage in different light-search behaviors to locate prey. These include phototaxis, where light is restricted to an area and movements towards the light source are guided by retinal input^65,66^ or dark photokinesis, where illumination is completely lost and locomotion is strongly increased^36,37^, being largely driven by non-retinal deep-brain photoreceptors expressing the light-sensitive pigment, melanopsin^38^. Recent findings however, show that increased larval locomotor activity during darkness is not random and undirected, as implied by the definition of photokinesis, but rather structured and resembles an area-restricted local search in a first phase followed by a more outward-directed roaming search to efficiently detect light sources^39^. With increasing age, decreasing yolk and under dark conditions, search strategy behavior becomes more important for the larvae and likely causes individuals to activate and strengthen their hard-wired program. This possibly explains our finding that the intra-individual consistency is highest under dark conditions and with advanced larval age under unfed conditions.

We performed our tests during 5 to 7 days post fertilization, as we aimed to find conditions for consistency in a standard well plate and for ages that are frequently used for zebrafish behavior studies. In addition, we chose this period to be able to test under unfed conditions, to avoid introducing another variable through feeding behavior. Food can introduce confounding factors in a high-throughput testing set up for drugs, pollutants or other perturbations, so identifying specific predictable periods for behavior testing without food is preferable^67,68^. To avoid potential feeding state related behavioral changes we stopped our tests at 7 dpf, although larvae can survive up to 10 days without food, with daily decreasing chances of survival^69^. It would, however, be interesting to investigate whether the observed consistency persists until adulthood, since adult zebrafish activity levels have also been shown to be consistent over several days^70^ and whether social interactions among the individuals could influence the consistency of this behavior. For some fish species, such as the Amazon molly (*Poecilia formosa*)^42^ or the mangrove killifish (*Kryptolebias marmoratus*) direct social experience did not influence the repeatability of behavioral measures in individuals, including exploration, boldness and aggression, despite the importance of social interactions in these species^71^. Yet in other species (e.g. guppy, *Poecilia reticulata*^72^; rainbow trout, *Oncorhynchus mykiss*^73^ and cichlid, *Neolamprologus pulcher*^74^), the social environment was shown to affect consistent individual behaviors, such as foraging, shoaling, boldness and aggression, and the development of animal personalities^75^.

Variability in zebrafish locomotor activity has previously been reported to decrease and locomotor activity to become more stable in the afternoon between 13.00 and 15.30^11^, a result that we could not replicate with our data. In fact, during dark intervals we found that the variability was moderately higher in the afternoons at 5 and 7 dpf. A possible explanation for this discrepancy is the difference in protocols as well as light conditions used between our study and the one performed by MacPhail *et al*.. When testing for time of day effect MacPhail *et al*. kept their larvae under infrared light in constant darkness throughout the period of testing, i.e. from 10.00 to 15.30, which might have resulted in less variability and more stability of the larvae’s locomotor activity when tested in the afternoon. In contrast, in our study, larvae were maintained in normal light conditions between the two test points within one day in order to mimic natural circadian light conditions and rhythms as closely as possible. Our data also revealed a high intra-individual consistency between morning and afternoon locomotor activity for all days tested and under all light conditions. This within-day consistency allows researchers interested in acute effects to record the baseline activity before the manipulation and thus calculate the effects more precisely.

By 5 dpf, zebrafish larvae perform a repertoire of simple sensorimotor behaviors that operate on characterized and accessible neural circuits^36,76–78^. For example, exposure to abrupt acoustic stimuli elicits a startle response, an evolutionary conserved and stereotyped yet modifiable behavior. Previous research has shown that zebrafish larvae habituate to a startle response. Best *et al*. demonstrated that zebrafish larvae (7 dpf) exhibit frequent reduction in response to a series of acoustic stimuli^79^, whilst wild-type larvae at 5 dpf have also been shown to rapidly reduce their startle response initiation and stereotypically habituate by more than 80% when exposed to a series of acoustic stimuli^80^. Additionally, Randlett *et al*. reported that zebrafish larvae habituated to a series of 1 second dark flashes each with a 60 s ISI, and that different components of the dark flash behavioral response, e.g. probability and latency, can be habituated separately by operating on different loci within the neuronal circuit^81^. In our study though, the larvae’s startle response was very inconsistent and unpredictable, for both the dark flash and tapping stimulus. There was one exception, with the responses to the first tapping stimulus showing moderate correlation between day 6 and 7. This correlation weakened over the 4 stimuli potentially as a result of inter-individual differences in startle response habituation, where some larvae habituated to the stimulus while others did not. Such individuality in habituation was reported for the acoustic startle response by Pantoja *et al*. who showed that the degree of habituation, despite being diverse, is stable and heritable for an individual^34^.

Although our data indicates occasional occurrences of habituation, consistency for an individual, as seen in previous studies, was not apparent. This may be due to the differing startle protocols used in the different studies, such as the inter-stimulus intervals lasted from 1 second^79,80^ to 5 seconds^34^ in the other studies, while in our study it was 90 seconds, which is much less likely to induce habituation. Another reason why we are not able to see consistency in the startle responses in general may be the small well sizes used in our setup which may have limited the response. Padilla *et al*. showed that the activity of 6 dpf larvae under dark and light periods was significantly higher for larvae kept in 24 well plates compared to 48 or 96 well plates^14^. Therefore, potentially the size of the well was reducing the activity of the fish resulting in the startle response being lost or masked. In addition, observations of the larvae’s response to the startle showed that whilst some swam towards the center, the wall edge stopped others (Supp. Video 1). This could suggest that occasionally the wall could be inhibiting the full startle response, which in turn could be preventing us from seeing intra-individual consistency in the startle responses.

Our thorough analysis of morphology and heart rates revealed values that were within the range of those reported in the literature^82–84^ and additionally revealed two interesting facts: a decrease of heart rates at 7 dpf, as well as a decrease in swim bladder size at 7 dpf. Concerning the heart rates, it is common to many fish species that the heart rate peaks right after hatching and then slows down whilst the fish matures. This drop in resting heart rate is thought to be due to the onset of inhibitory nervous control of the heart by the vagus nerve shortly after hatching^85,86^. Furthermore, the decrease in swim bladder size could be because of a change in shape, as the developing swim bladder undergoes a process to eventually form a double-chambered swim bladder in the adult stage^84,87^. When looking at the links between the behavior and morphology of fish, previous studies have shown strong links. A study by Hawkins and Quinn (1996) investigated if morphological and physiological traits explained variations in critical swimming speed and found that the best swimming fish had longer caudal regions than the poorer swimmers^88^. Larger brown trout have also been shown to have greater stamina and attained higher swimming speeds than smaller fish, along with maximum swimming speed additionally correlating with fish size^89^. Studies with juvenile zebrafish have shown that individual body size had a strong effect on the activity-boldness relationship, where smaller fish were bolder and less active while larger fish were more cautious and active^90^. In addition, differences in zebrafish parental swimming stamina led to differences in cardiac and metabolic output of their offspring in early stages around 10 dpf^83^. In our study, despite strong intra-individual consistency, no such links between behavioral movements and morphological or cardiophysiological parameters were observed under any of the conditions measured. This difference between our data and this literature may be as a result of our study being conducted over development. However, other literature is in line with our findings in terms of the lack of this link, as for the bluegill sunfish (*Lepomis macrochirus*), neither boldness nor locomotion activity correlated to the body size or condition of the fish^91^. Importantly, locomotor activity was shown to be independent of weight and body length in adult zebrafish^70^. Therefore, the link between morphology and behavior may be dependent on the age, conditions and type of behavior investigated.

Scientific research is constantly under intense scrutiny, specifically for the occurrence of irreproducible and non-comparable findings. In particular, high-throughput behavioral tests frequently result in inconsistent findings, which researchers attribute to poor quality science and non-standardized protocols^9–13^. However, this problem also strongly links to the lack of understanding of the variability of basic behavioral patterns, as fish fundamentally change their swimming behavior over time^1–7,92^. Considering such changes along with the variability would allow the design of a statistically more robust experiment yielding relevant and reliable results. The data from our study is important in helping the development and generation of reproducible zebrafish behavioral data, such as those generated in neurotoxicity or drug discovery tests^8,93–96^. Our results indicate that the behavioral locomotor machinery of an individual, although still under maturation, becomes stable over those key larval stages that are frequently used for testing (6–7 dpf). This stability manifests after the establishment of the beat-and-glide swim mode and strengthens when the locomotor network calls upon during darkness-induced exploration, and results in a consistent period at 6 dpf under dark intervals. In addition, measures between morning and afternoon showed a high intra-individual consistency for all days and light conditions tested. The revealed intra-individual consistency provides some basis to improve the estimation of acute behavioral effects of substances and other types of treatments through pre-post exposure measurements. In addition, this study has highlighted areas where high levels of inter- and/or intra- individual variability occur, specifically for the response to a startle stimulus and morphological and cardiophysiological features of the larvae which should be accounted for when used in future studies. This data not only highlights the need to consider the design and experimental setup/conditions but also provides a basis to allow future studies to account for variability when using zebrafish locomotor behavior. This in turn could help to encourage the inclusion of variation as an additional endpoint, as some external challenges do not change the mean of the population but lead to a change in variation. This might have important ecological consequences for the population to adapt to new environmental conditions, because variation creates the basis for adaptation. Thus, the inclusion of variation might provide new insights into the understanding of an individual’s and population’s response to an external challenge.

## Material and Methods

### Zebrafish husbandry

Mixed wildtype (WM) zebrafish (*Danio rerio*), originally obtained from crosses between AB, Tübingen and a pet shop population (OBI, Leipzig, Germany) were maintained under standard conditions^97^ in accordance with the Swiss animal protection law. Adult fish were maintained in a mixed sex Mass Embryo Production System (Aquatic Habitats^®^, Pentair Aquatic Eco-systems, USA), linked to a recirculating flow-through supplied with a mixture of tap and desalted water (1:1) at 26 ± 1 °C, under a 14:10 h light:dark cycle. Oxygen saturation in the system was 86.03 ± 6.8%, pH was 8.1 ± 0.2, conductivity was 354 ± 29.6 µS/cm^2^. Adult fish were fed twice daily to satiation from a combination of dry flakes (Tetra, Germany) and live food (*Artemia nauplii*). Group crosses resulted in larvae for the behavior trials, with eggs collected approximately 1 hour post-fertilization (hpf). Eggs were then rinsed and incubated in artificial freshwater that was aerated to oxygen saturation (according to ISO-7346/3 guideline^98^) prior to use and unfertilized eggs were removed during the blastula stage as described by Kimmel *et al*.^99^. Fish were raised in petri dishes (approx. 50 per dish) until required for behavioral experiments in an incubator with the same light and temperature conditions as described above, using ISO artificial freshwater, which was changed every 24 hours. All experiments were carried out in accordance with relevant animal protection guidelines, while all experiments involving larvae were approved by the Cantonal Veterinary Office Zürich, Switzerland under the license ZH168/17.

### Behavioral tracking and recording

At 4 dpf, larvae were randomly distributed across 48 well plates (Greiner Bio-One, Austria), with 1 larva added to an individual well containing 500 µl of fresh ISO water. Larvae were moved in the morning and were returned to the housing incubator until the following day, at which point behavioral experiments were initiated. Behavior was recorded using the DanioVision Observation Chamber (v. DVOC-0040T; Noldus, Netherlands), which consists of both a Gigabit Ethernet video camera fitted with infrared and white-light sources, and a transparent multi-well plate holder. The camera output was fed into a standard PC system with the EthoVision XT13 software (version 13.0.1220, Noldus, Netherlands) which created videos for later analysis of locomotor activity. All behavior experiments were carried out in a temperature-controlled room maintained at 26 ± 1 °C.

Larvae were subjected to different protocols to allow for thorough assessment of their movement. The first protocol consisted of an acclimation period of 20 minutes in light, to allow the fish to adjust to the Noldus set up and to allow for their baseline movement to settle. This was followed by a 20 minute measurement of spontaneous swimming behavior (referred to as “spontaneous” throughout the manuscript). This was then followed by, alternating dark and light periods each lasting 10 minutes (2x dark periods, referred to as “dark intervals”; 2x light periods, referred to as “light intervals”). Immediately following this protocol, the larval response to a short pulse of darkness was recorded. Larvae were left for 90 seconds in light before being subjected to a 1 second pulse of darkness, which was repeated four times, with an inter-stimulus-interval (ISI) of 90 seconds applied to allow fish to settle down and reach the baseline in between each stimulus. This ISI was selected as it was shown by Pantoja *et al*.^34^ to be sufficient to avoid habituation of fish to an acoustic stimulus. The same pattern was used for the tapping protocol, however in this instance the stimulus was produced using the inbuilt DanioVision Tapping Device (Noldus, Netherlands)^40^ which produces an acoustic vibrational stimulus. The swimming protocol was recorded at 30 frames/s while the startle protocols were recorded at 60 frames/s. All of the behavioral protocols were run at 9 am and 2 pm, on 5, 6 and 7 dpf, with same protocols applied at each point (Supp. Fig. 1). Between 9 am and 2 pm measurements, well plates were maintained under defined light conditions in the testing room to most closely mimic a normal diurnal cycle. The experiment, spanning 3 days, was repeated 3 times at different periods, and due to mortalities arising through handling, a sample size of n = 132 larvae were produced for analysis (Rep 1: n = 46, Rep 2: n = 41 and Rep 3: n = 45). There was no effect of the replicate on the behavior of the larvae for any of the conditions tested (spontaneous: χ^2^ (1) = 0.18, p = 0.672; dark intervals: χ^2^ (1) = 0.07, p = 0.787; spontaneous: χ^2^ (1) = 0.83, p = 0.361). In addition, there was no effect of location of the fish within the plate for any of the conditions tested (e.g. spontaneous swimming- well: χ^2^ (47) = 44.82, p = 0.563; column: χ^2^ (1) = 0.16, p = 0.693; row: χ^2^ (5) = 4.79, p = 0.442).

### Heart rate and morphological measurements

At 4 pm on each of the 3 days, immediately after the afternoon behavior measurements, videos were recorded of each larva for the measurement of heart rates and morphometric parameters. Larvae were anesthetized with 160 mg/L of ethyl 3-aminobenzoate methanesulfonate (MS222; Sigma-Aldrich), a level of anesthesia previously shown to have no influence on zebrafish heart rates^82^. A 15 second video of each larva in the lateral position was then captured, at 30 frames/s, using a Basler acA2000-165 µM camera, mounted on a Leica S8APO stereo microscope. Videos were recorded using the Media Recorder 4 software (version 4.0; Noldus, Netherlands). After a suitable video had been collected, larvae were immediately moved into a bath of 100% air saturated ISO water to allow for recovery from the anesthetic procedure. Recovered larvae were then returned to their original locations, albeit in a new 48 well plate containing 500 µl of ISO water per well. The well plate was returned to the incubator until the next behavioral measurements were recorded.

From the collected videos, the heart rate and morphology parameters were measured using DanioScope Software (version 1.2.206; Noldus, Netherlands). After manually defining the heart area in each video, the software calculated the number of beats per minute using a power plot spectrum of the frequencies extracted from an activity signal. For each larva, 3 heart rate measurements were taken for each day from the same video and the average of these was taken as the heart rate for that larva on that day. The morphological parameters measured using DanioScope software were body length (from nose to tip of the tail; mm) and swim bladder size (from the lateral view of the larvae; mm^2^). To minimize the possibility of biases being introduced during image processing, the same calibration profile was used to analyse all images.

### Data and statistical analysis

Tracking of the fish by the EthoVision software was carried out from the videos in a non-live tracking mode, allowing a static subtraction of the background, thereby reducing tracking artefacts. To prevent biases introduced through data processing, the same processing settings were applied to data from different days, times and replicates. To characterize the swimming behaviors during the spontaneous, dark and light intervals, the activity and radial index were calculated^41^. The activity index represents the percentage of movement by each larva over one-second intervals and was calculated using a threshold of 2.00 to 1.75 cm/s. The radial index indicated the position of the larva within the well and was calculated by dividing the distance of a larva from the center of the well (mm; calculated in the EthoVision software) by 5.725 mm, the radius of each well. The coefficient-of-variation of a larva’s activity was calculated from the standard deviation of its activity, divided by its mean (average) activity.

For the startle response strength, the distance moved per second was used instead of the activity index. This did not affect the significance or trends that were observed with the activity index (Supp. Table 2), but rather allowed for a more detailed view of the specific movements of the fish. The period one-second post stimulus was taken to analyze the distance moved by the fish while performing a startle response. To calculate the strength of each response with respect to spontaneous movement, a baseline for each fish was determined before each stimulus. The distance moved per second during a 40 second period, 10 seconds before the startle stimulus, was averaged and SD added to give baseline level of distance moved before the stimulus. This period was selected to ensure the larva’s activity was no longer affected by the previous stimulus. The response strength was then calculated by subtracting the baseline from the distance moved during the startle stimulus. Using this baseline, the habituation index was calculated from ratios between the first strength response and either the second, third or fourth strength response. The sum of all ratios was taken as the habituation score for that larva. This score was calculated for each individual for each day and time.

All statistical analyses were performed using ‘RStudio’ software (version 1.1.453, USA). To carry out the correlation analysis, the Pearson’s correlation coefficient, r, was calculated and reported, and the p-values to test these correlations were obtained from a two-sided t-test. A linear regression model was used to draw lines of best fit for the scatter plots. To assess differences between group and between experimental conditions, analysis of variance models were carried out using the minimum adequate model approach, where model simplification using F-test occurred based on analysis of deviation. A Tukey HSD multiple comparison analysis was carried out on significant models to test between group effects. Linear mixed/effect analysis were used to test for positional effects of the well, as well as to evaluate effects from the different repeats. Well location, column, row, edge and repeat were entered as fixed effects, while individual larva were entered as random effects. P-values were obtained by likelihood ratio tests of the full model, comparing the effect in question against the equivalent model in the absence of effect. For all tests, data were considered statistically significant when p < 0.05.

## Supplementary information

Supplementary Information

Supplementary Video 1

## Data Availability

The datasets generated and analyzed during the current study are available in the ERIC repository, https://data.eawag.ch/.
