# Supplementary material for: Emergence of consistent intra-individual locomotor patterns during zebrafish development
Source: Sci Rep. 2019 Sep 20;9:13647. doi: 10.1038/s41598-019-49614-y (PMC6754443; doi:10.1038/s41598-019-49614-y)
Supplement: Supplementary file 1 — Supplementary Information [file 41598_2019_49614_MOESM1_ESM.pdf]

Supplementary Information: Emergence of consistent intra-individual locomotor patterns during zebrafish development

Jennifer A. Fitzgerald, Krishna Tulasi Kirla, Carl P. Zinner, Colette M. vom Berg

Pages

FIGURES

Supp. Fig. 1.....S2

Supp. Fig. 2 .....S3

Supp. Fig. 3.....S4

Supp. Fig. 4 .....S5

Supp. Fig. 5 .....S6

Supp. Fig. 6 .....S7

TABLES

Supp. Tab. 1.....S8

Supp. Tab. 2 .....S9

Supp. Tab. 3.....S10

Supp. Tab. 4 .....S11

Supp. Tab. 5 .....S12

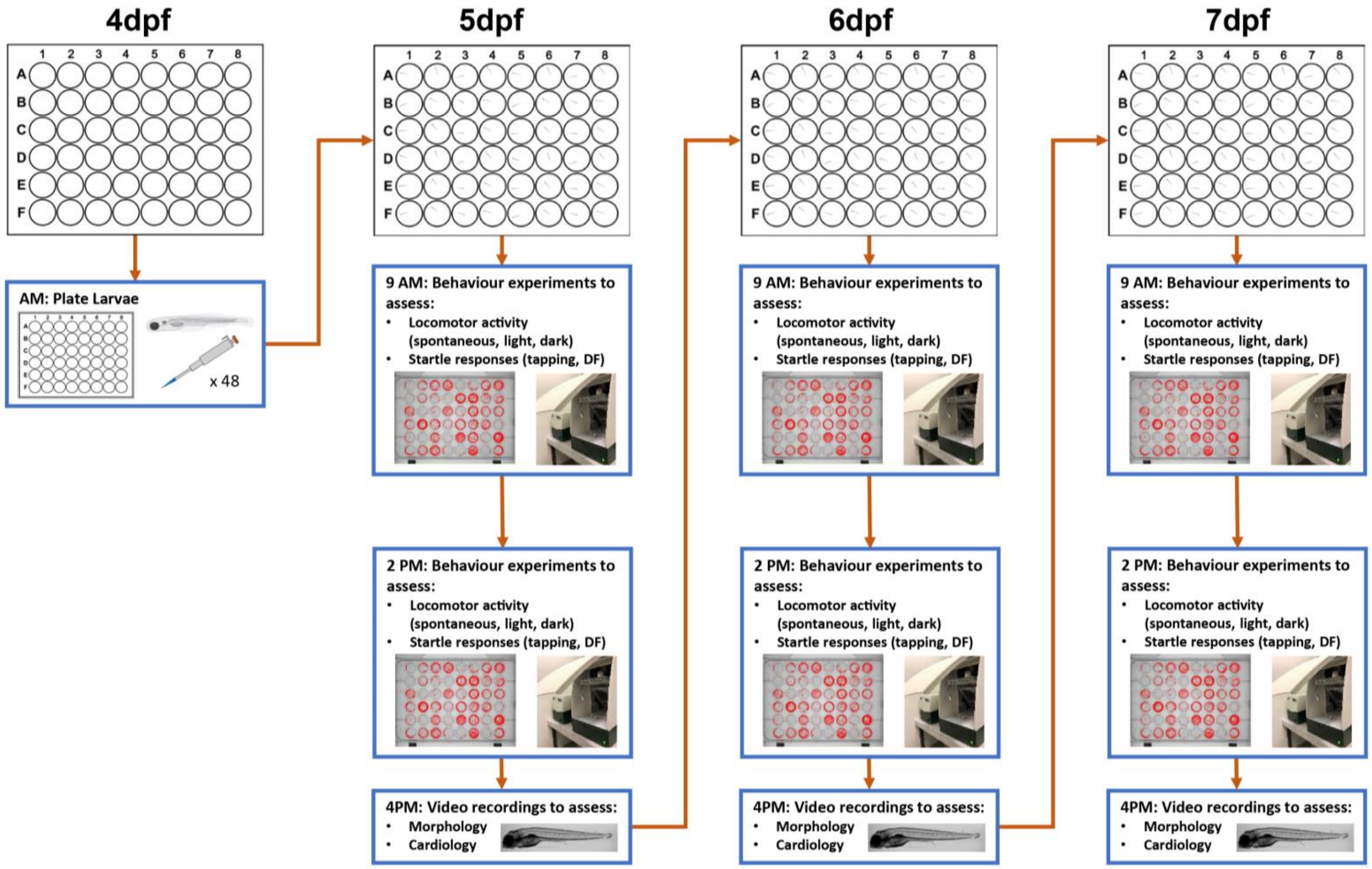

Supp. Fig. 1: Scheme detailing how the experiments were arranged and carried out in this study. One batch of 48 larvae was used in the same behavioral experiments (spontaneous, light intervals, dark intervals, tapping and dark flash (DF)), which were carried out on three consecutive days (5, 6, 7 days post fertilisation (dpf)) at two time points (9am and 2pm), with additional video recordings at 4pm for heart rate and morphology assessment. Three replicates of this scheme were carried out.

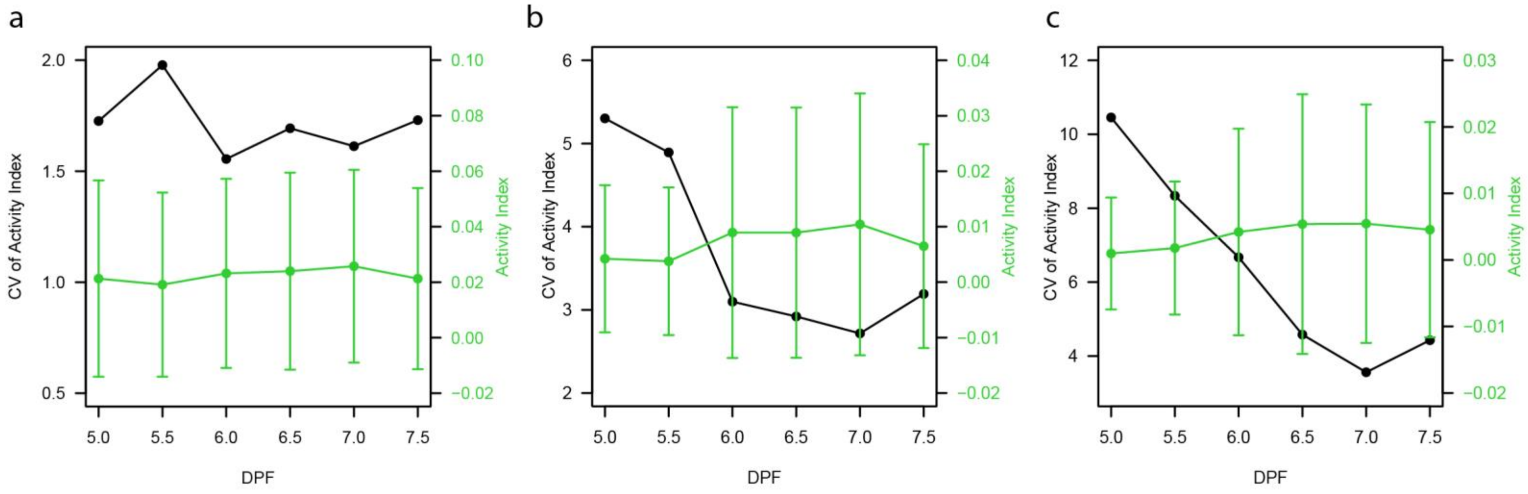

Supp. Fig. 2: Plots showing the median coefficient of variation (CV) (black) for each individual larva (n = 132) and the average activity index ± standard deviation (green) over the different days (days post fertilization; dpf) and daytimes, for (a) dark intervals, (b) spontaneous and (c) light intervals.

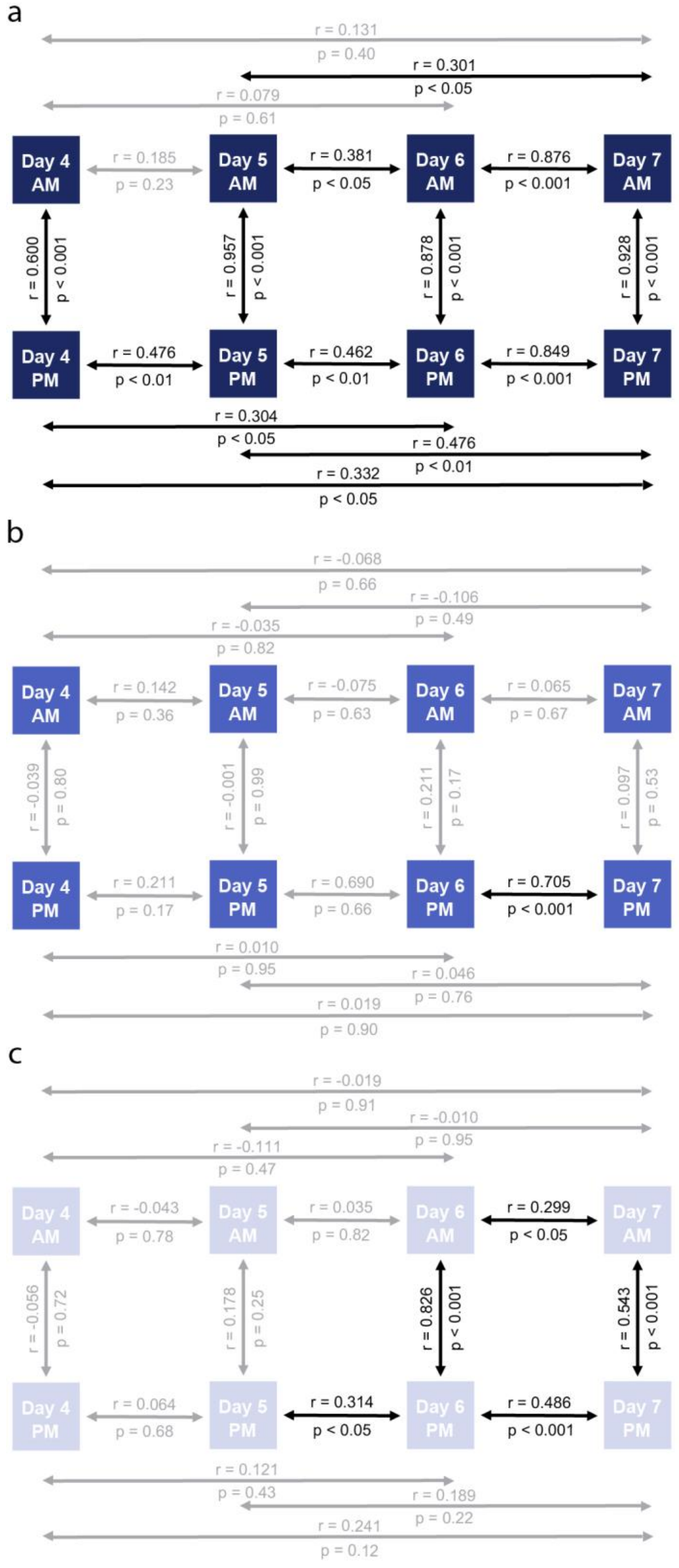

Supp. Fig. 3: Schematics representing the correlations of the activity index for the 4-day study between different days and time of days for each of the conditions studied, (a) dark intervals, (b) spontaneous and (c) light intervals. All correlation coefficients derived from Pearson's correlations with respective p values.

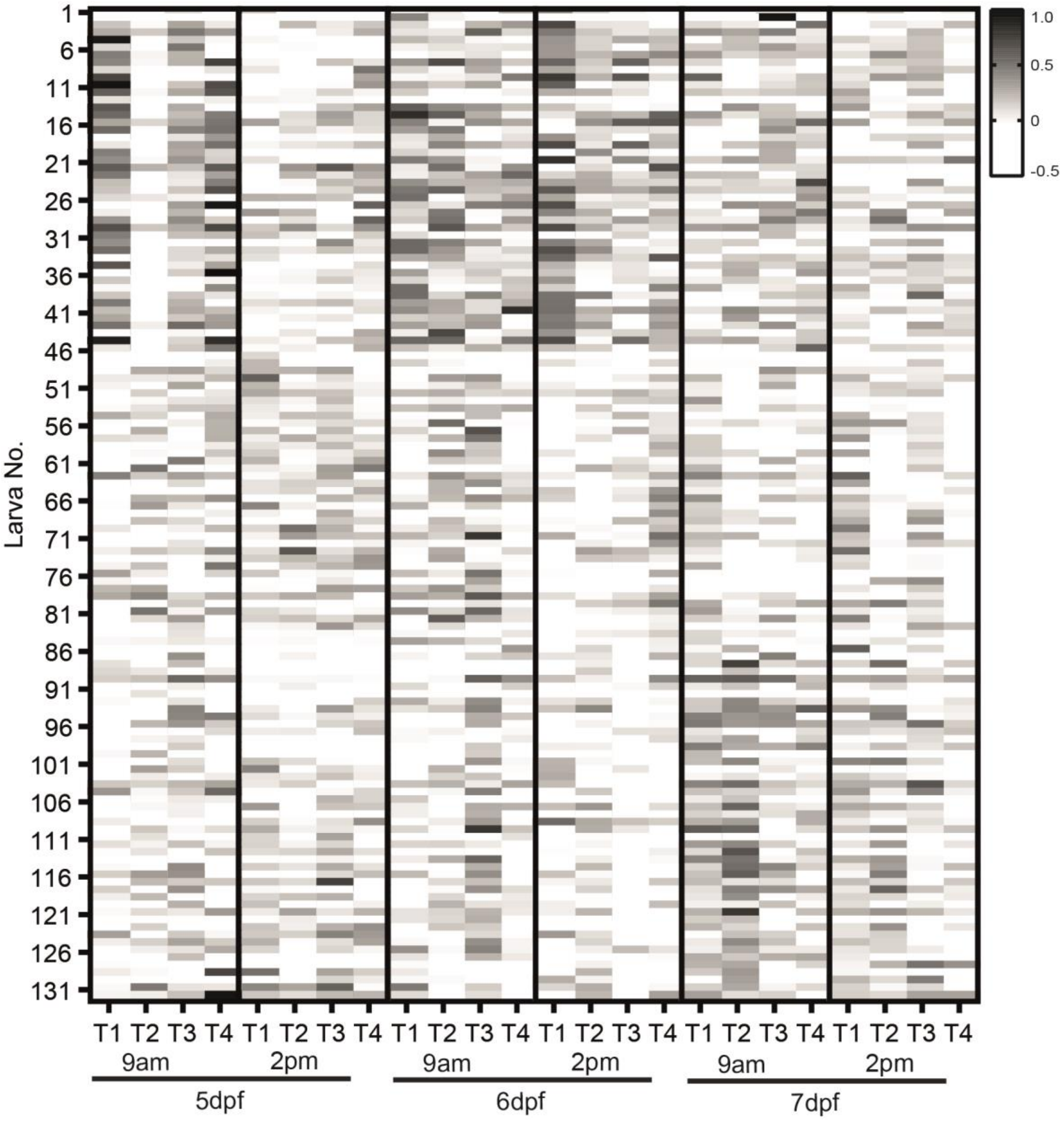

Supp. Fig. 4: Heat map representing the change in distance moved with respect to the baseline of each individual larva for all time points and days measured in response to each of the dark flash stimulus (S1-S4). White represents no response to the stimulus, with the grey scale darkening in a linear scale depending on the strength of the response.

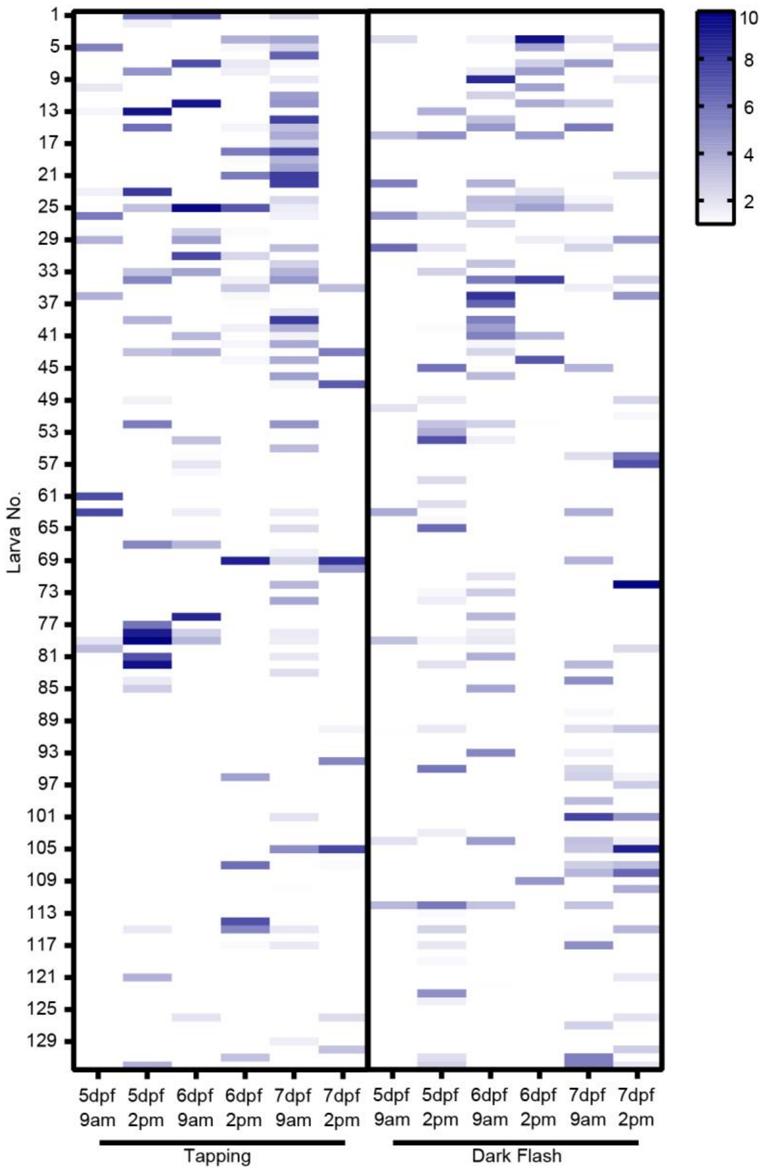

Supp. Fig. 5: Heat map showing the habituation index of the 132 individual larvae for all time points and days measured in response to the tapping (left) and dark flash (right) stimuli. White represents no habituation, with blue darkening in a linear scale depending on the strength of habituation calculated.

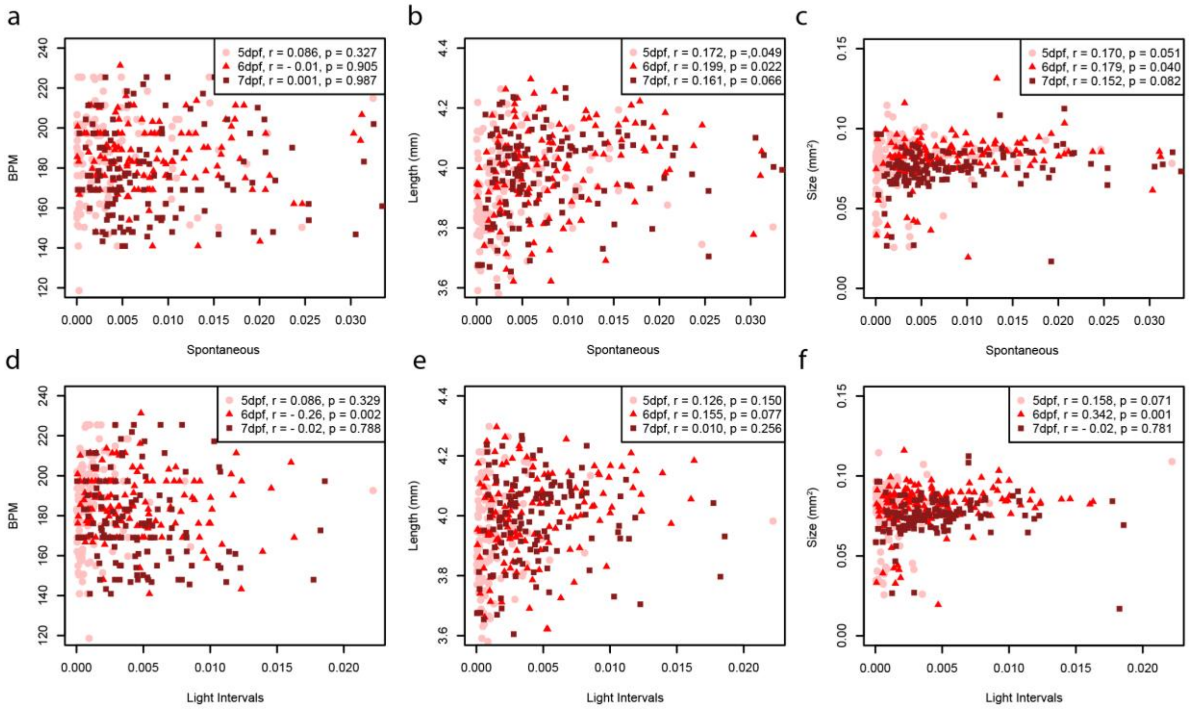

Supp. Fig. 6: Comparison of the individual larvae's (a, d) heart rate (in beats per minute, BPM), (b, e) body length (in mm) and (c, f) size of swim bladder (in mm^2) to their respective average activity during spontaneous (a, b, c) and light intervals (d, e, f), with each day plotted on each plot (5 days post fertilization (dpf): circle, 6 dpf; triangle and 7 dpf; square). Statistics on the plots represent the Pearson's correlation coefficient and respective p value.

Supp. Tab. 1: Table of correlations between the activity and radial index for all conditions tested (spontaneous, dark intervals and light intervals) for all days and times tested. All correlation coefficients were derived from Pearson's correlations, with color scale based on the r value (blue positive correlation and yellow negative correlation). Significant correlations are denoted by the bold font when $p < 0.05$.

	Day	Time	Activity v Radial	
			r	p value
**Spontaneous**	5	9am	0.14	0.116
		2pm	-0.02	0.846
	6	9am	**0.21**	**0.014**
		2pm	**0.24**	**0.005**
	7	9am	**0.31**	**3.54** $\times 10^{-4}$
		2pm	**0.23**	**0.009**
**Dark Intervals**	5	9am	**0.37**	**1.22** $\times 10^{-5}$
		2pm	**0.35**	**3.65** $\times 10^{-5}$
	6	9am	**0.34**	**5.59** $\times 10^{-5}$
		2pm	**0.32**	**1.82** $\times 10^{-4}$
	7	9am	**0.21**	**0.015**
		2pm	**0.21**	**0.018**
**Light Intervals**	5	9am	0.04	0.638
		2pm	0.08	0.350
	6	9am	0.17	0.057
		2pm	0.09	0.305
	7	9am	-0.01	0.894
		2pm	0.06	0.480

Supp. Tab. 2: Table of comparisons of the output from the correlation analysis when using the activity index compared to distance moved (cm). Values given are the r values from Pearson's correlations, with color scale based on that r value (blue positive correlation and yellow negative correlation). Significant correlations are denoted by the bold font when $p < 0.05$.

Experiment	Day	Time	Activity Index		Distance Moved	
			r	p value	r	p value
Tapping v Dark Flash	5dpf	9am	**0.247**	**4.28×10^{-4}**	**0.338**	**7.43×10^{-5}**
		2pm	0.057	0.52	0.060	0.50
	6dpf	9am	**0.176**	**0.04**	**0.437**	**1.60×10^{-7}**
		2pm	**0.192**	**0.03**	**0.276**	**0.00**
	7dpf	9am	-0.117	0.18	**-0.226**	**0.01**
		2pm	0.046	0.60	**0.266**	**2.04×10^{-3}**
Tapping v Dark Intervals	5dpf	9am	**0.186**	**0.03**	0.131	0.13
		2pm	-0.007	0.93	0.032	0.71
	6dpf	9am	-0.064	0.47	0.032	0.72
		2pm	0.159	0.07	0.053	0.55
	7dpf	9am	0.007	0.94	0.057	0.51
		2pm	-0.093	0.29	**-0.177**	**0.04**
Tapping v Spontaneous	5dpf	9am	0.029	0.74	-0.011	0.90
		2pm	0.138	0.11	**0.382**	**6.30×10^{-6}**
	6dpf	9am	0.041	0.64	0.037	0.67
		2pm	0.145	0.10	0.030	0.74
	7dpf	9am	-0.138	0.12	0.038	0.67
		2pm	0.002	0.98	-0.111	0.21
Tapping v Light Intervals	5dpf	9am	0.174	0.05	-0.024	0.79
		2pm	0.158	0.07	**0.234**	**0.01**
	6dpf	9am	-0.060	0.49	-0.121	0.17
		2pm	0.155	0.08	**0.195**	**0.03**
	7dpf	9am	-0.013	0.88	0.019	0.83
		2pm	-0.109	0.21	-0.120	0.17
Dark Flash v Dark Intervals	5dpf	9am	0.045	0.61	0.129	0.14
		2pm	0.156	0.07	0.014	0.87
	6dpf	9am	0.067	0.45	0.081	0.36
		2pm	0.061	0.49	0.035	0.69
	7dpf	9am	**0.232**	**0.01**	**0.202**	**0.02**
		2pm	0.126	0.15	0.061	0.49
Dark Flash v Spontaneous	5dpf	9am	0.166	0.06	0.151	0.08
		2pm	-0.010	0.91	-0.047	0.60
	6dpf	9am	**0.213**	**0.01**	**0.213**	**0.01**
		2pm	-0.036	0.68	-0.090	0.30
	7dpf	9am	0.144	0.10	**0.260**	**2.63×10^{-3}**
		2pm	0.165	0.06	0.093	0.29
Dark Flash v Light Intervals	5dpf	9am	0.029	0.74	0.000	1.00
		2pm	0.018	0.84	0.010	0.91
	6dpf	9am	-0.070	0.43	-0.077	0.38
		2pm	-0.002	0.98	-0.059	0.50
	7dpf	9am	0.158	0.07	0.165	0.06
		2pm	-0.011	0.90	-0.004	0.96

Supp. Tab. 3: Table of correlations between the startle responses (tapping, dark flash, onset and offset) for all days and times tested. Values given are the r values from Pearson's correlations, with color scale based on that r value (blue positive correlation and yellow negative correlation). Significant correlations are denoted by the bold font when $p < 0.05$.

			Tapping						Dark Flash						Offset						Onset					
			5		6		7		5		6		7		5		6		7		5		6		7	
			9am	2pm	9am	2pm	9am	2pm	9am	2pm	9am	2pm	9am	2pm	9am	2pm	9am	2pm	9am	2pm	9am	2pm	9am	2pm	9am	2pm
Tapping	5	9am		0.127	**0.263**	-0.080	0.106	0.005	**0.338**	-0.105	0.134	0.106	-0.083	-0.050	0.131	0.155	0.079	-0.032	0.108	-0.053	0.060	0.046	0.151	0.060	0.126	0.006
		2pm			**0.395**	0.092	**0.230**	0.044	**0.188**	0.060	**0.216**	0.166	-0.275	-0.025	-0.033	-0.008	0.126	-0.076	**0.172**	-0.026	-0.119	-0.041	-0.027	-0.064	**0.172**	-0.007
	6	9am				**0.251**	**0.423**	-0.031	**0.278**	-0.082	**0.437**	**0.293**	**-0.242**	-0.103	0.048	-0.073	0.085	-0.157	0.012	-0.160	-0.074	-0.037	0.140	-0.027	**0.245**	0.025
		2pm					**0.270**	**-0.404**	0.144	-0.112	**0.318**	**0.276**	-0.027	-0.105	-0.131	-0.018	-0.004	0.093	0.093	0.043	-0.054	-0.085	0.031	0.012	0.142	0.152
	7	9am						-0.106	**0.482**	-0.103	**0.486**	**0.563**	**-0.226**	-0.111	0.022	0.080	0.091	-0.122	0.062	-0.005	-0.046	0.120	0.122	0.050	**0.223**	0.178
		2pm							**-0.268**	0.091	-0.145	**-0.320**	0.018	**0.266**	0.130	0.061	0.092	-0.150	0.092	0.035	-0.130	0.064	0.135	-0.081	-0.070	**-0.207**
Dark Flash	5	9am								-0.098	**0.435**	**0.475**	**-0.456**	-0.056	**0.314**	**0.214**	0.097	-0.022	0.096	0.087	0.088	0.143	0.145	0.084	**0.202**	0.162
		2pm									-0.076	0.022	0.051	-0.053	0.132	0.129	0.153	0.139	0.063	0.046	-0.111	-0.094	0.012	0.072	-0.069	0.060
	6	9am										**0.475**	-0.156	-0.072	0.151	0.044	**0.266**	0.061	0.166	0.153	0.026	**0.202**	0.156	0.091	**0.221**	0.166
		2pm											**-0.486**	-0.013	0.063	**0.188**	0.146	0.064	**0.244**	**0.216**	0.003	0.147	**0.277**	0.125	**0.206**	**0.181**
	7	9am												**0.213**	**0.229**	**0.200**	**0.208**	**0.421**	**0.278**	**0.332**	-0.010	0.004	0.028	-0.159	-0.025	-0.053
		2pm													0.089	**0.203**	**0.180**	0.156	**0.297**	**0.201**	0.069	**0.222**	-0.012	0.020	-0.063	-0.120
Offset	5	9am														**0.356**	**0.297**	**0.215**	0.166	**0.334**	-0.038	-0.030	0.102	0.047	0.028	-0.021
		2pm															**0.228**	**0.228**	**0.220**	**0.224**	0.048	**0.184**	-0.042	-0.082	-0.079	0.019
	6	9am																**0.334**	**0.426**	**0.392**	-0.077	0.043	**0.187**	0.015	0.015	0.035
		2pm																	**0.292**	**0.492**	-0.089	0.112	0.153	0.059	-0.057	0.047
	7	9am																		**0.517**	0.032	0.016	0.029	0.080	0.011	0.051
		2pm																			-0.115	0.064	0.047	0.033	0.030	-0.021
Onset	5	9am																				**0.324**	**0.193**	**0.208**	0.118	**0.181**
		2pm																					0.127	0.095	**0.250**	**0.197**
	6	9am																						**0.278**	**0.377**	**0.231**
		2pm																							**0.226**	**0.342**
	7	9am																								**0.263**
		2pm																								

Supp. Tab. 4: Table of correlations between the startle responses (tapping, dark flash, onset and offset) and the different conditions (spontaneous, dark intervals and light intervals) for all days and times tested. Values given are the r values from Pearson's correlations, with color scale based on that r value (blue positive correlation and yellow negative correlation). Significant correlations are denoted by the bold font when $p < 0.05$.

			Dark Intervals						Light Intervals						Spontaneous					
			5		6		7		5		6		7		5		6		7	
			9am	2pm	9am	2pm	9am	2pm	9am	2pm	9am	2pm	9am	2pm	9am	2pm	9am	2pm	9am	2pm
Tapping	5	9am	0.131	0.152	**0.192**	-0.035	0.059	0.034	-0.024	0.014	0.025	-0.214	-0.060	-0.052	-0.011	0.076	-0.033	**-0.186**	0.038	-0.138
		2pm	-0.063	0.032	0.026	**-0.213**	-0.028	-0.025	**0.190**	**0.234**	0.038	**-0.342**	-0.089	-0.144	**0.185**	**0.382**	-0.083	**-0.208**	-0.076	-0.166
	6	9am	-0.047	0.039	0.032	-0.148	0.077	0.055	-0.062	0.058	-0.121	**-0.322**	-0.063	-0.002	0.112	0.153	0.037	**-0.283**	0.023	-0.069
		2pm	-0.191	-0.104	-0.043	0.053	0.127	0.166	-0.163	-0.130	-0.112	**0.195**	**0.290**	**0.328**	0.064	0.052	0.040	0.030	**0.227**	0.153
	7	9am	-0.110	0.012	0.035	**-0.198**	0.057	0.032	0.006	0.094	-0.057	**-0.302**	0.019	0.104	0.066	0.156	0.060	**-0.297**	0.038	-0.109
		2pm	-0.151	-0.078	**-0.233**	**-0.308**	**-0.176**	**-0.177**	-0.058	-0.023	-0.099	**-0.283**	-0.113	-0.120	-0.049	0.000	-0.025	-0.159	-0.111	-0.111
Dark Flash	5	9am	0.129	0.120	0.166	-0.171	0.052	-0.019	0.000	0.138	-0.020	**-0.262**	-0.044	-0.096	0.151	**0.222**	0.107	-0.142	0.087	-0.111
		2pm	-0.061	0.014	-0.015	0.029	0.012	0.007	0.121	0.010	-0.042	0.035	-0.020	-0.068	-0.099	-0.047	-0.076	-0.053	-0.121	-0.012
	6	9am	-0.013	0.067	0.081	-0.082	0.111	0.045	0.089	0.153	-0.077	-0.119	0.027	0.007	0.103	0.131	**0.213**	-0.143	0.116	-0.098
		2pm	0.084	0.155	**0.187**	0.035	**0.254**	**0.184**	0.027	-0.064	0.005	-0.059	0.105	-0.024	0.020	0.064	0.016	-0.090	**0.175**	0.042
	7	9am	0.045	-0.016	0.130	**0.204**	**0.202**	0.099	-0.023	-0.149	-0.073	**0.263**	0.165	0.030	-0.143	-0.061	0.063	**0.284**	**0.260**	0.177
		2pm	0.160	0.080	-0.072	-0.026	0.133	0.061	-0.167	-0.112	-0.030	-0.030	0.051	-0.004	-0.108	-0.135	-0.052	0.107	0.127	0.093
Offset	5	9am	0.136	0.033	0.162	-0.008	0.142	0.012	-0.005	0.100	0.008	-0.098	-0.154	**-0.326**	0.166	0.202	**0.179**	0.090	-0.022	**-0.172**
		2pm	0.150	0.110	0.064	-0.016	0.067	-0.007	-0.059	0.023	-0.053	-0.059	-0.082	-0.133	-0.022	0.115	-0.094	-0.051	0.022	-0.153
	6	9am	-0.005	0.012	0.157	-0.001	**0.203**	0.109	0.021	-0.060	-0.078	-0.008	0.009	-0.186	-0.080	0.090	**0.217**	-0.096	0.131	-0.042
		2pm	-0.048	-0.063	0.017	0.143	**0.224**	0.123	-0.011	**-0.196**	-0.134	**0.303**	0.132	-0.070	-0.079	-0.016	0.027	**0.262**	0.162	0.055
	7	9am	0.002	-0.067	0.053	0.016	**0.285**	**0.200**	0.061	-0.042	-0.078	0.084	**0.200**	0.023	0.058	-0.020	0.026	-0.011	**0.270**	0.140
		2pm	-0.098	-0.085	0.042	0.089	**0.345**	**0.244**	0.007	-0.087	-0.066	**0.193**	**0.186**	-0.081	0.143	0.126	0.150	0.159	**0.251**	**0.193**
Onset	5	9am	**0.497**	**0.466**	**0.278**	**0.314**	**0.305**	**0.340**	0.031	0.128	0.067	0.049	0.171	**0.275**	-0.055	-0.082	0.094	0.056	**0.245**	**0.225**
		2pm	**0.340**	**0.397**	0.111	-0.001	0.151	0.066	-0.004	0.112	-0.010	-0.072	0.107	-0.027	0.012	0.104	0.024	0.063	**0.197**	-0.005
	6	9am	**0.240**	**0.262**	**0.512**	**0.309**	**0.395**	**0.355**	**0.172**	0.067	-0.022	0.111	0.056	-0.015	0.032	0.070	**0.218**	0.027	0.114	0.073
		2pm	0.110	0.110	0.103	**0.317**	**0.204**	**0.244**	0.008	-0.009	-0.085	0.114	-0.095	0.079	-0.071	-0.135	0.056	0.064	0.031	0.152
	7	9am	0.119	**0.228**	**0.314**	0.105	**0.364**	**0.282**	0.079	0.136	0.007	-0.020	0.103	-0.013	**0.189**	**0.193**	**0.214**	-0.046	**0.188**	-0.056
		2pm	-0.058	-0.051	0.127	0.089	**0.186**	0.153	-0.025	-0.048	-0.024	0.040	0.039	0.093	-0.027	-0.022	0.136	0.011	0.066	0.007

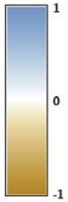

Supp. Tab. 5: Table of correlations between days for each morphological parameter (resting heart rate (BPM; beats per minute), body length and swim bladder size). All correlation coefficients were derived from calculating Pearson's correlations, with color scale based on that r value (blue positive correlation and yellow negative correlation). Significant correlations are denoted by the bold font when $p < 0.05$.

Morphological Parameter	DPF Comparison	r	p value
BPM	5 v 6	**0.48**	**4.41 $\times 10^{-9}$**
	5 v 7	**0.61**	**1.50 $\times 10^{-14}$**
Body Length	5 v 6	**0.79**	**4.96 $\times 10^{-29}$**
	5 v 7	**0.64**	**2.16 $\times 10^{-16}$**
Swim Bladder Size	5 v 6	**0.63**	**1.06 $\times 10^{-15}$**
	5 v 7	**0.32**	**1.97 $\times 10^{-4}$**
